# Zoonotic Poxviruses Associated with Companion Animals

**DOI:** 10.3390/ani1040377

**Published:** 2011-11-17

**Authors:** Danielle M. Tack, Mary G. Reynolds

**Affiliations:** 1 Epidemic Intelligence Service, Centers for Disease Control and Prevention, Atlanta, GA 30333, USA; 2 Poxvirus and Rabies Branch, Centers for Disease Control and Prevention, Atlanta, GA 30333, USA; E-Mail: nzr6@cdc.gov

**Keywords:** poxvirus, zoonoses, companion animals, orthopoxvirus, parapoxvirus, monkeypox, cowpox, orf

## Abstract

**Simple Summary:**

Contemporary enthusiasm for the ownership of exotic animals and hobby livestock has created an opportunity for the movement of poxviruses—such as monkeypox, cowpox, and orf—outside their traditional geographic range bringing them into contact with atypical animal hosts and groups of people not normally considered at risk. It is important that pet owners and practitioners of human and animal medicine develop a heightened awareness for poxvirus infections and understand the risks that can be associated with companion animals and livestock. This article reviews the epidemiology and clinical features of zoonotic poxviruses that are most likely to affect companion animals.

**Abstract:**

Understanding the zoonotic risk posed by poxviruses in companion animals is important for protecting both human and animal health. The outbreak of monkeypox in the United States, as well as current reports of cowpox in Europe, point to the fact that companion animals are increasingly serving as sources of poxvirus transmission to people. In addition, the trend among hobbyists to keep livestock (such as goats) in urban and semi-urban areas has contributed to increased parapoxvirus exposures among people not traditionally considered at high risk. Despite the historic notoriety of poxviruses and the diseases they cause, poxvirus infections are often missed. Delays in diagnosing poxvirus-associated infections in companion animals can lead to inadvertent human exposures. Delays in confirming human infections can result in inappropriate treatment or prolonged recovery. Early recognition of poxvirus-associated infections and application of appropriate preventive measures can reduce the spread of virus between companion animals and their owners. This review will discuss the epidemiology and clinical features associated with the zoonotic poxvirus infections most commonly associated with companion animals.

## 1. Introduction

Poxviruses have played a large role in human history. The most infamous of the poxviruses is variola, the causative agent of smallpox. Descriptions of smallpox can be found in Chinese texts from 4th Century AD and pox-like scars found on Egyptian mummies suggest the disease may have existed as early as 2nd millennium BC [1]. Smallpox was the first disease to have an effective vaccine and variola was the first human virus to be eradicated [2]. It was Jenner's observations regarding the zoonotic infection with ‘cowpox virus’ that led to the discovery of modern vaccinology [1]. Vaccinia virus, the virus now used for smallpox vaccination, was the first animal virus to be seen via electron microscopy and the first to be used as a vector for carrying foreign genes into animals [2].

Despite their historic notoriety, infections caused by poxviruses are commonly missed in today's world. In Europe, humans, cats and pocket pets have fallen ill with cowpox, but rarely is poxvirus infection initially suspected by the patient or the clinician. In 2003, the United States experienced an outbreak of monkeypox involving both humans and animals. Eight people became symptomatic before the first case was reported to public health authorities [3]. One could argue that these infections are missed because they are rare. However, parapoxviruses such as orf are considered ubiquitous and cause infection in livestock worldwide. Lesions are readily recognized in herds by experienced farmers, but hobbyists and less experienced producers might fail to take appropriate measures to prevent ongoing infections within the herd and to themselves. In humans, poxvirus infections are neglected zoonoses and are undoubtedly under-reported, so the true impact of these diseases remains unknown.

The term companion animal is often used synonymously with ‘pet’ which implies affection and frequent interaction. Traditionally this has included dogs and cats; however, rodents and other small mammals are also often kept as pets, and in the last few decades exotic animals and livestock have become more common as companion animals. These changes in pet demographics along with increased importation of exotic animals have resulted in poxviruses occurring in areas outside their normal geographic range and in certain high-risk human occupational groups. Therefore, it is important that pet owners and human and animal clinicians develop a heightened awareness for poxvirus infections and understand the risk associated with specific companion animals. Delays in diagnosing animal infections can lead to inadvertent human exposures and delays in human diagnosis could result in inappropriate treatment or delayed recovery. Also, early recognition of poxvirus infections is important for implementation of appropriate measures aimed at preventing virus spread. This review will focus on those zoonotic poxviruses most likely to be associated with companion animals and discuss what is currently known about poxvirus pathogenesis and diagnosis in general, as well as the epidemiology and clinical features associated with these zoonotic infections.

## 2. General Features of Poxviruses

The skin is the primary portal of entry for most poxviruses, however respiratory and mucosal routes have been associated with orthopoxvirus infections. Poxviruses are epitheliotropic; most infections are self-limiting and produce localized lesions that progress through macular, papular, vesicular and pustular stages over several weeks. Ultimately the lesion forms a crust and often leaves a characteristic “pock” scar for which the virus was originally named. Generalized infections occur depending on the species of virus and host. In generalized infections, virus spread is thought to occur through the regional lymphatics to the bloodstream, resulting in a primary viremia. The virus then multiplies in reticuloendothelial organs followed by a secondary viremia and seeding to other sites especially the skin [4,5].

The tropism of poxviruses is thought to be a function of host genes and immune response post cell entry, as opposed to virus binding and entry as is seen for most viruses [6]. Zoonotic poxviruses are represented in at least three genera of poxviruses: Orthopoxvirus, Parapoxvirus, and Yatapoxvirus. In general, most zoonotic infections are primarily acquired through direct contact with an infected animal which results in cutaneous lesion(s) at the site of contact; however, there is evidence that some cases have occurred via mucosal routes or following contact with infected fomites. Table 1 summarizes the features of zoonotic poxviruses addressed in this review.

## 3. Orthopoxviruses

Many orthopoxviruses, including cowpox, monkeypox, and vaccinia, have a broad host range. Although the reservoir species for most of the zoonotic orthopoxviruses remain undetermined, the most likely candidates are sylvatic rodents which normally have little interaction with people, but may interact with animals commonly kept as pets. Domestic cats are efficient predators and though cowpox has been reported in a variety of species in Europe and Western Asia, clinical infections are primarily seen in cats. Rodents kept as pets have also been implicated in human disease outbreaks [7-11]. In the United States, pet prairie dogs were the source of a monkeypox outbreak in people that was later traced to the importation of African rodents [3]. In the European Union, virus infected fancy rats that were purchased in pet stores across France, the Netherlands, and Germany were responsible for over 30 human cases of cowpox [7-12]. Not only have pet owners been affected by orthopoxviruses, those involved in veterinary care or husbandry of animals have also been affected [3,13-18]. Because of the role companion animals play in human infections of cowpox and monkeypox the epidemiology and clinical features of these viruses will be discussed further.

### 3.1. Cowpox

**Figure 1:**
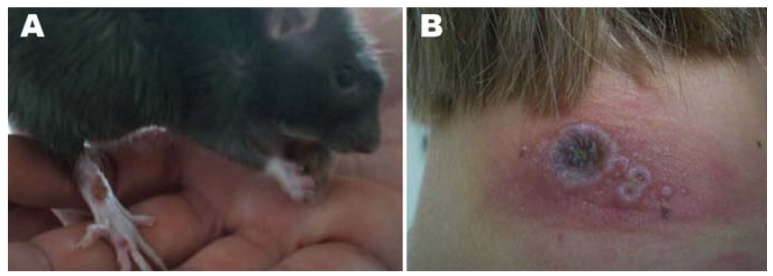
Clinical presentation of cowpox lesions on rats and humans during an outbreak in Germany, 2009. (**A**) Pet rat with lesions on the right hind limb; and (**B**) Neck lesions on a girl [10] (Photos originally printed in Emerging Infectious Diseases by Campe, H.; *et al.*).

Today cowpox is known to infect a broad range of species throughout Europe and Western Asia. Wild rodents are considered the reservoir, but the exact species is unknown. In the United Kingdom there is strong evidence supporting bank and field voles as reservoir species with wood mice and other rodents capable of maintaining the virus [19-21]. Although human infection was once associated with cattle, today it is predominately associated with domestic cats. DNA isolates from cats in England are closely related to early cattle and human isolates from the same region [14,22]. Feline infections usually occur during the autumn months, which correlate with peak rodent population size and activity; therefore, it is thought that they become infected while hunting rodents, through a bite, scratch or possibly ingestion [23,24]. Pet rats and other non-domestic animals, such as monkeys and elephants have also been associated with human infection (Figure 1) [8-11,25-29]. Transmission to humans likely occurs via direct contact with an affected animal resulting in implantation of the virus into non-intact skin or mucus membranes [13]. Children and those involved in animal care appear to be at most risk [14].

Most persons ultimately diagnosed with cowpox virus infection present to their physician with a single, painful pustular lesion located on the hands or face and complain of “flu-like” symptoms. The patient's usually have a history of owning a cat that ‘gifts’ them with dead rodents or they have had close contact with an ill cat or rodent. Cowpox lesions tend to be painful throughout the life of the lesion, are large, ulcerative with inflammation and edema and the crust it forms is thick, hard and black. Local lymphadenopathy and systemic symptoms such as pyrexia, lethargy, sore throat and general malaise are common and often severe enough for people to miss school or work. Infections are typically self-limiting as most people recover in 6–8 weeks [13,14]. Mucosal lesions have also been reported [30]. Generalized infections, some fatal, have only been reported in those with predisposing factors such as eczema, atopic dermatitis, and Darier's disease [13,14,31,32]. Clinically cowpox can be mistaken for parapox, herpes, or anthrax.

Infections in animals can be nearly asymptomatic or, at the other end of the spectrum, can result in death. Clinically significant cowpox has been recognized most often in *Felidae sp*, especially the domestic cat, but has also been described in the domestic dog, rats, monkeys, elephants, and other animals [9,25,26,29,33-38]. Most cats present with a generalized papular or vesicular rash which may have started as a single ‘bite-like’ lesion around the head, neck or forelimb approximately 1–2 weeks prior. Bacterial dermatitis is often suspected initially. Approximately 20% of infected cats will develop oral lesions, and 20% will have a mild upper respiratory tract infection with serous to mucopurulent nasal and ocular discharge similar to what is seen with calicivirus infections and feline herpes. Fatal outcomes in domestic cats have primarily been associated with underlying disease. However, underlying disease, such as feline immunodeficiency virus, does not always indicate a poorer prognosis [23]. Clinical signs in pet rats are primarily upper respiratory signs and death; however, skin lesions, particularly on the paws, have been reported in fancy rats (Figure 1) [8-10,39]. Of the three reports in dogs, all infections have been single, ulcerated lesions [33,34]. Disease tends to be more severe and often results in death non-domestic animals such as lions (*Panthera leo*), anteaters (*Mymecophaga tridactyla*), ocelots (*Leopardus pardalis*), cheetahs (*Acinonyx jubatus)*, monkeys and elephants [27-29,35-37,40].

### 3.2. Monkeypox

Monkeypox is endemic to tropical rainforests of central and western Africa and rodents, possibly squirrels, are the likely reservoir candidates. The cumulative annual incidence of human disease is considered low and person-to-person transmission has been documented [41-46]. When person-to-person spread occurs it is thought to be the result of either direct contact or respiratory droplets. The primary mode of zoonotic transmission in endemic settings is somewhat unclear [44,47]. Genetic analysis has identified two distinct clades of monkeypox virus—western and central African [48-50]. Western clade isolates are thought to be less virulent in humans and less apt to spread person-to-person than central African variants of monkeypox virus. The virus isolated during the 2003 outbreak in the United States was most closely linked to western clade isolates. Disease was first recognized in humans that had handled or cared for ill prairie dogs at home. The outbreak was eventually linked to several species of rodents imported from western Africa for the exotic pet market, including Gambian rats. The exotic species had been warehoused or transported with prairie dogs destined for sale as pets [3]. Transmission from animals to people appeared to be through direct contact with animal lesions or respiratory secretions or indirect contact with contaminated bedding [51].

During the outbreak people presented with a generalized rash and lymphadenopathy which was proceeded by a 2–4 day prodrome of fever, headache, backache, lethargy and general malaise. Similar to what is seen in central and western Africa [3,44,47]. Unique to the outbreak were cases that presented similar to those with cowpox infections—nodular primary lesions around the margins of a bite or scratch with focal hemorrhagic necrosis. However, unlike typical cowpox presentations, a disseminated rash followed in immunocompetent individuals. These individuals also suffered from more severe systemic disease and had a compressed incubation period [3,51]. Common differential diagnoses include chickenpox, secondary yaws, and syphilis.

**Figure 2:**
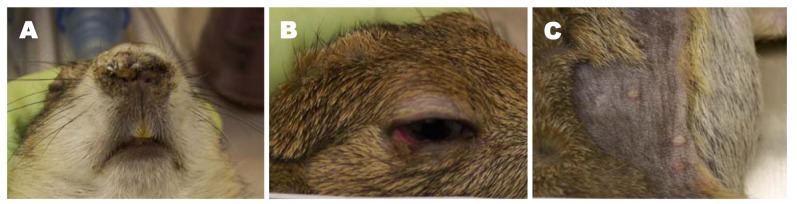
Clinical signs of monkeypox in experimentally prairie dogs. (**A**) Nasal discharge; (**B**) Blepharitis and (**C**) Disseminated skin lesions (Photos associated with the study available at http://libproxy.cdc.gov:2073/science/article/pii/S0042682210001650).

During the US monkeypox outbreak pet prairie dogs presented with lethargy, anorexia, lymphadenopathy, blepharitis with ocular discharge, and upper respiratory signs such as cough, sneezing, and nasal discharge which eventually lead to pneumonia (Figure 2(a,b)). Based on this presentation infections were originally suspected to be tularemia or plague. Occasionally a papular rash was present and sudden death was also noted (Figure 2(c)) [3,52,53]. Evidence of monkeypox exposure was found in other rodents housed at the same facility as animals originating from the African shipment; however, clinical signs were not described [54]. Experimental infections conducted on a variety of rodents, including African dormice and ground squirrels, describe anorexia, lethargy, and death as the most common signs of disease and varying degrees of respiratory signs [55-57]. Transmission amongst rodents appears to be similar to what is seen with people, occurring either via direct contact or respiratory droplet [52]. Experimental infection has also been described in non-human primates and is similar to what is seen in people, except without the prodrome [58-60].

## 4. Parapoxviruses

In the United States from 2001 to 2006 there was 50% increase in the number of households keeping livestock species (sheep, goat, cows) as pets [61]. These animals may not be kept solely for companionship, but identifying them as pets implies they are being kept for reasons not directly related to the owner's livelihood. Urban farms are also on the rise as both a source for local food production and for raising livestock as a hobby, especially goats. Therefore, parapoxvirus infections can no longer be classified as just an occupational hazard or confined to rural areas. The epidemiology and clinical features of human and small ruminant infections will be discussed further.

### 4.1. Orf

Orf is also known as contagious pustular dermatitis, contagious ecthyma, soremouth, or scabby mouth in sheep and goats. It is considered enzootic wherever goats and/or sheep are raised and sporadic infections have occurred in cats and dogs [62,63]. Transmission occurs when the virus comes into contact with a non-intact epithelial surface either through direct contact or contact with fomites. The virus is robust in dry environments and can survive for months or years. Despite a rigorous host immune response reinfection commonly occurs as a result of a short-lived humoral response [64,65]. The incidence and prevalence of infection in humans is undefined. One rural practice in Walesattempted to quantify the prevalence in their patients and found 73 of 251 respondents reported having orf at least once, a quarter of which did not consult a doctor [66]. In sheep and goats morbidity is generally high and mortality rarely exceeds 1%, although rates have been reported as high as 10% in lambs and 93% in kids [67,68]. Disease in animals and humans is often seen in spring which corresponds to the lambing and kidding seasons [69-71]. Disease in people is commonly seen in farmers, veterinarians, and abattoir worker, but has also been identified in those that slaughter or prepare sheep and goats carcasses for personal consumption, as occurs, for example, in conjunction with the Muslim holy day Eid-al-Adha, Feast of Sacrifice [65,72-78].

**Figure 3:**
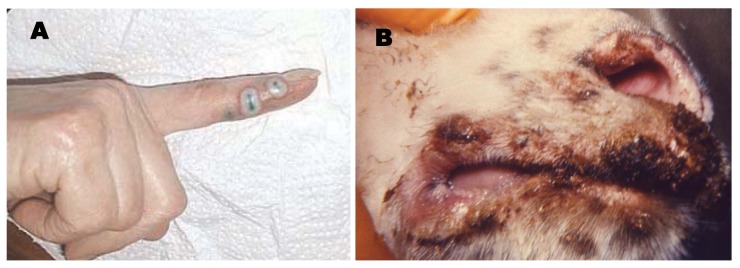
Clinical presentations of orf lesions in a human and a sheep. (**A**) Lesions at the site of a bite from a sheep on day 19 postinoculation [83]; (**B**) Proliferative lesions involving the lips and muzzle of a goat infected with orf. (Photo A courtesy of Scottish Medical Journal Copyright 2011 Royal Society of Medicine Press; Photo B provided by Callis, J.J.; and Mahy, B.W.J. courtesy of CDC Public Health Image Library http://phil.cdc.gov/phil/home.asp).

People typically present with a solitary, non-healing lesion on their hands, occasionally on the face or axilla, and when asked report contact with sheep, goats, or associated husbandry items [71,73,79-82]. The vesicular stage has a characteristic “target” appearance with a red center, white ring and red halo and progresses to a weeping nodule (Figure 3(a)). The nodule eventually dries creating small black dots on the surface and as it heals, papillomas develop over the lesion surface. Resolution typically occurs in 6 weeks with little or no scarring [69]. Complications such pain, fever, lymphangitis, and erythema multiforme have been reported [69,83,84]. Very rarely generalized disease and large progressive lesions occur (referred to as ‘giant’ orf); these cases are most often associated with an immunocompromising condition in the host [63,70,85-90]. Unlike orthopoxvirus lesions, parapoxvirus lesions tend to be proliferative rather than ulcerative.

In small ruminants, orf occurs most commonly in juvenile animals; although in a naïve herd, large numbers of adults can also be affected. Animals generally present with lesion around the commissures of the lips (Figure 3(b)). Lesions are usually large, proliferative, and have 2–4 mm raised crusts, but on mucosal surfaces they are often ulcerated. In severe cases the gingiva, dental pad, palate, and/or tongue are involved leading to anorexia and weight loss [65,91]. Lesions have also been identified on the ears and tails (thought to be associated with ear tagging or tail docking) as well as on the udders of nursingewes and does [92-94]. Severe generalized infections characterized by disseminated proliferative lesions, pneumonia, and arthritis has been described in goats [95,96]. Prompt diagnosis can be complicated when there is clinical disease in goats, but sheep located on the same premises are asymptomatic [95,97].

### 4.2. Other Parapoxviruses

Other recognized parapoxviruses known to cause human infection are found in cattle (bovine papular stomatitis virus and pseudocowpox virus), deer, seals and sea lions [76,98-104]. Lesions in people do not differ from what is seen with orf. Therefore, animal exposure history is important for initial clinical differentiation [82,98,99,105,106]. Bovine papular stomatitis and pseudocowpox are clinically indistinguishable and require PCR techniques for differentiation. However, lesions do appear to have a predilection for specific sites and signalment. Bovine popular stomatitis lesions primarily occur on the muzzle, nose, and hard palate oral mucosa of young feedlot cattle and can cause an ulcerative esophagitis, while pseudocowpox primarily affects the teats, udder, and perineum in dairy cows [107-112]. In pinnipeds, lesions do not have a predilection for a specific anatomical region, whereas in deer, lesions have been reported on the head and neck [101,113,114].

## 5. Diagnosis and Treatment of Poxviruses

### 5.1. Diagnosis

**Figure 4:**
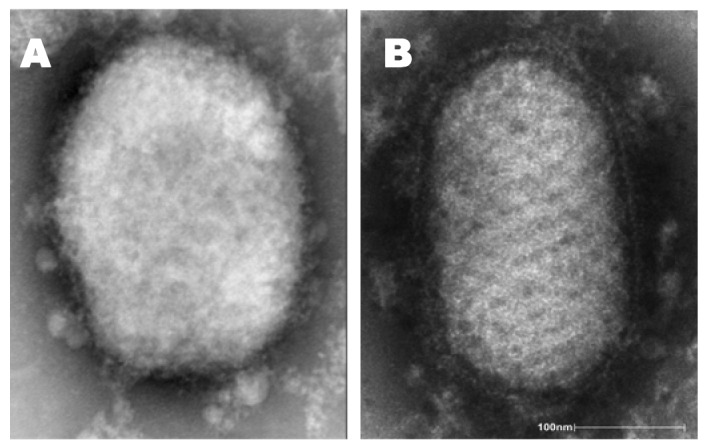
Electron microscope photos of (**A**) orthopox (monkeypox) and (**B**) parapox (orf) viruses (Photo A provided by Humphrey, C.D.; Morehead, T.; and Regnery, R.; Photo B taken by Goldsmith, C.; and provided by Likos, A.; both images courtesy of CDC Public Health Image Library http://phil.cdc.gov/phil/home.asp).

Diagnosis of some poxvirus infections can be made based on clinical features and case history alone. However, laboratory testing is needed to confirm infection. Because large quantities of virus can be found within lesions, vesicle fluid, crusts, or tissue biopsy are preferred as diagnostic samples. Electron microscopy and histopathology can also be helpful for confirming a diagnosis as poxviruses have distinctive morphology—large (approximately 300 nm diameter), box or ovoid shape, and outer membrane protrusions (creating a textured appearance)—and replicate in the cytoplasm of host cells [4,115,116]. Parapoxviruses exhibit a unique oval shape and criss-cross pattern of the outer membrane (Figure 4(a,b)) [115].

Cowpox can sometimes be differentiated from other orthopoxvirus infections by the presence of large, cytoplasmic, eosinophilic A-Type inclusion bodies [116,117]. Serologic tests are available; however, their utility is limited due to cross-reactivity within the various poxvirus genera [118]. PCR techniques and electron microscopy (EM) are the most common methods for rapid identification of poxvirus-associated infections and for virus species determination [119-123]. Although PCR and EM are available for confirming zoonotic poxvirus infections, additional tests that are both rapid and reliable are needed at the point of patient care so that physicians can manage patients appropriately and avoid severe complications of infection in children and the immunocompromised [122].

### 5.2. Treatment and Prevention

In general, poxvirus infections are self-limiting and treatment is primarily supportive care. Antibacterial therapy is only warranted when secondary infection is present. Promising antivirals, developed under the auspices of bioterrorism preparedness, are currently under investigation, but there has been little consideration of their potential application to the prevention and treatment of poxvirus-associated zoonoses. Experimental evidence suggests that compounds with direct antiviral effects such as Cidofovir and CMX-001 and others that inhibit viral egress such as ST-246 may be effective in treating orthopoxvirus infections [124-128]. Cidofovir and imiquimod creams have also been used in treating human cases of orf [85,89,129-131]. Unfortunately, similar therapeutics are not used in animals; however, there is experimental evidence of their effectiveness in small ruminants [132,133]. The acceptability of using these antivirals in livestock is unknown, but their use in companion animals and other high-value animals (zoo animals) could be explored.

Vaccination is also available for prevention in some instances. Smallpox vaccine is considered protective against orthopoxvirus infections and is recommended for laboratory personnel working with monkeypox, cowpox, vaccinia, and variola [134]. In animals, the modified vaccinia virus Ankara (MVA) smallpox vaccine is routinely used in elephants for protection against cowpox [28,135]. Vaccines for orf are available for sheep and goats and are primarily used to reduce the severity of clinical signs [136,137]. Both the smallpox and orf vaccines are live viruses which result in an infectious lesion at the site of vaccination, therefore, care must be taken to avoid autoinoculation or inadvertent transfer of the virus to a susceptible host [138,139]. The virus used to vaccinate for orf is non-attenuated; therefore, vaccination can result in contamination of the environment similar to natural infection [140]. Since the agent survives for long periods of time in the environment, it is important that equipment is thoroughly cleaned and properly disinfected. The quaternary ammonium compound Lysoform casa has been shown to be effective for disinfecting equipment even in the presence of organic material [141].

## 6. Conclusions

Many factors play into the emergence or reemergence of zoonotic disease and recent reports of poxvirus infections demonstrate that human behavior often plays a role. A plausible scenario for a future outbreak could involve transportation or abandonment of an infected companion animal(s) resulting in the release of poxvirus into a new environment. Our current understanding of poxvirus epidemiology suggests a need to more efficiently identify and control of these diseases in novel populations.

Public health efforts that focus on increasing poxvirus awareness in animal owners could have a positive impact on behaviors that can reduce the risk of infection, including hand washing after handling an animal, applying quarantine when introducing new animals to a flock or herd, wearing gloves when manipulating an animal's oral cavity or when a cut is present on the hand, and seeking veterinary care for an ill pet. Early clinical recognition of poxvirus infections in animals can prevent virus spread, and heightened awareness in animal owners may lead to early health seeking behaviors, which will in turn optimize opportunities for clinical interventions.

With increased human and animal movement across oceans the possibility for future outbreaks of zoonotic poxviruses is very real. This observation suggests a need for the development of therapeutics and biologics for both animals and humans. Antivirals are probably not a valid option in animals due to costs and concerns for resistance, but vaccines can play an important role. Research that focuses on vaccines that provide protection without contaminating the environment are key to protecting both human and animal health. Until approved therapies become available, early recognition of infection is important for preventing the spread of virus. Through heightened awareness and appropriate prevention measures, poxvirus infections can be minimized both in people and in their companion animals.

## Figures and Tables

**Table 1:** Select zoonotic poxviruses and their associated distribution and clinical features.

**Genus**	**Virus**	**Geographic location**	**Reservoir**	**Primary zoonotic source**	**Clinical features in animals**	**Clinical features in humans**
Orthopoxvirus	Cowpox	Europe & Western Asia	small rodents (voles & wood mice)	domestic cats	single bite like lesion on head or extremity that develops into a generalized papular rash; upper respiratory signs	painful, large, ulcerative lesion on hand or face with inflammation and edema; thick, hard, black crust; flu-like symptoms
	Monkeypox	Western & Central African rainforests	UNK; suspect rodents	rodent species	sudden death; upper respiratory signs, anorexia, lymphadenopathy, blepharitis; +/− generalized papular rash	2–4 day prodrome of headache and fever; generalized rash and lymphadenopathy; single nodule with focal hemorrhagic necrosis at innoculation site (hand)
Parapoxvirus	Orf	World-wide	sheep & goats	sheep & goats	large proliferative lesions with raised crust primarily around comissures and muzzle	Single or multiple lesions on upper extremities (especially hands) or face; vesicle has “target” appearance (red center, white ring, red halo), papillomas over surface prior to crusting
	Bovine Papular Stomatitis	World-wide	cattle	cattle	primarily young feedlot cattle ; lesions usually on muzzle, nose & hard palate; erosions & ulcers common	see Orf
	Pseudocowpox (Paravaccinia)	World-wide	cattle	cattle	primarily dairy cows; lesions usually on teats, udder & perineum	see Orf
